# Utilizing a contralateral hamstring autograft facilitates earlier isokinetic and isometric strength recovery after anterior cruciate ligament reconstruction: a randomised controlled trial

**DOI:** 10.1007/s00167-021-06491-1

**Published:** 2021-02-18

**Authors:** Christoffer von Essen, Alexander Hallgren, Björn Barenius, Karl Eriksson

**Affiliations:** 1 Department of Orthopaedics, Stockholm South Hospital, Karolinska Institute, Stockholm, Sweden; 2 Capio Artro Clinic, FIFA Medical Centre of Excellence, Sophiahemmet Hospital, Valhallavägen 91, 11486 Stockholm, Sweden

**Keywords:** ACL, ACL reconstruction, Contralateral, Hamstring

## Abstract

**Purpose:**

To compare muscle strength and patient reported outcomes following ACLR using a semitendinosus (ST) graft from the ipsilateral (IL) leg compared to a graft from the contralateral (CL) leg.

**Methods:**

One-hundred and forty patients with an ACL injury were randomized to IL or CL ACLR. Patients were assessed at 6, 12 and 24 months with isokinetic and isometric muscle strength measured using Biodex. Patient-reported outcomes and manual stability measurements were also recorded.

**Results:**

Patient-related outcomes improved over time for both groups with no significant differences between groups at any time point. No differences between groups in objective knee assessment scores or rerupture rates were found. The IL group was significantly weaker in knee flexion strength at all time points compared to the CL group, additionally the IL group did not recover flexor strength within 2 years.

**Conclusion:**

This study demonstrated that utilizing an ST graft harvested from the uninjured limb for ACLR facilitates early isokinetic and isometric strength recovery, with no significant adverse outcomes demonstrated in other measurements and therefore be performed to reduce the risk of long-term strength deficits in the injured leg

**Level of evidence:**

II.

## Introduction

In Sweden, approximately 8000 ACL injuries are reported each year, with approximately 4000 ACL reconstructions performed annually [2]. Although conservative treatment can be successful in the appropriate population, it is less likely to succeed in patients aiming to return to a high level of sporting activity.

The most widely used grafts for reconstruction of the ACL include the patellar tendon (BPTB), hamstring tendon (HS), and quadriceps tendon (QT). Studies comparing outcomes of ACLR with these grafts have not shown one to be clearly superior over the others with regards to knee stability [20, 21, 30, 32]. BTPB grafts have been shown to generate more donor site morbidity than both HT and QT grafts [1, 17, 26], however, there is no clear consensus regarding which graft achieves the best overall patient outcomes.

In Sweden, a hamstring graft using semitendinosus (ST) is utilized in 95% of ACLRs, mainly to minimize donor site morbidity [2]. However, it is fair to say that the perfect graft for ACLR does not exist.

There have been studies using BTPB graft from the contralateral noninjured leg for primary ACLR, and in the context of revision surgery, with good results [24, 25]. Additionally, Yasuda [33] performed a study using contralateral semitendinosus-gracilis (ST-G) grafts to distinguish morbidity attributable to graft harvest from the ACLR, and McRae et al. [16] have performed a randomized controlled trial using contralateral ST-G hamstring graft. Neither study identified any significant drawbacks or benefits associated with using an ST-G graft from the unaffected limb. To our knowledge, there are no studies assessing the use of an ST-graft only.

Studies have shown that the tendon harvest of ST does not compromise function and strength as much as harvesting both ST and G. As such, the technique of harvesting ST whilst preserving gracilis (G) has been recommended [12, 23, 34]. Activity related soreness rarely limits activity and has usually resolved by three months. Furthermore, studies have shown that some regrowth or scar formation of the tendon remnants occurs in a majority of cases [1, 12, 17, 23]. In theory, the use of a graft from the uninjured leg allows the injured limb to avoid additional damage in connection with the ACLR. Another potential advantage may be the early restoration of symmetrical hamstring strength, as a reduced strength ratio (Hamstring/Quadriceps ratio (H/Q)) may be a risk factor for ACL ruptures in females [9].

The objective of this study was to evaluate the outcomes of ACLR using a ST graft harvested from the contralateral leg, compared to ACLR with a ST graft harvested from the ipsilateral leg, in terms of flexion muscle strength and patient-reported outcomes. It was hypothesized that using a CL graft would facilitate earlier strength recovery.

## Materials and methods

The study was approved by the regional ethics committee at the Karolinska Institute, Stockholm Sweden (reference no. 2013/1398-31/2).

**Fig. 1:**
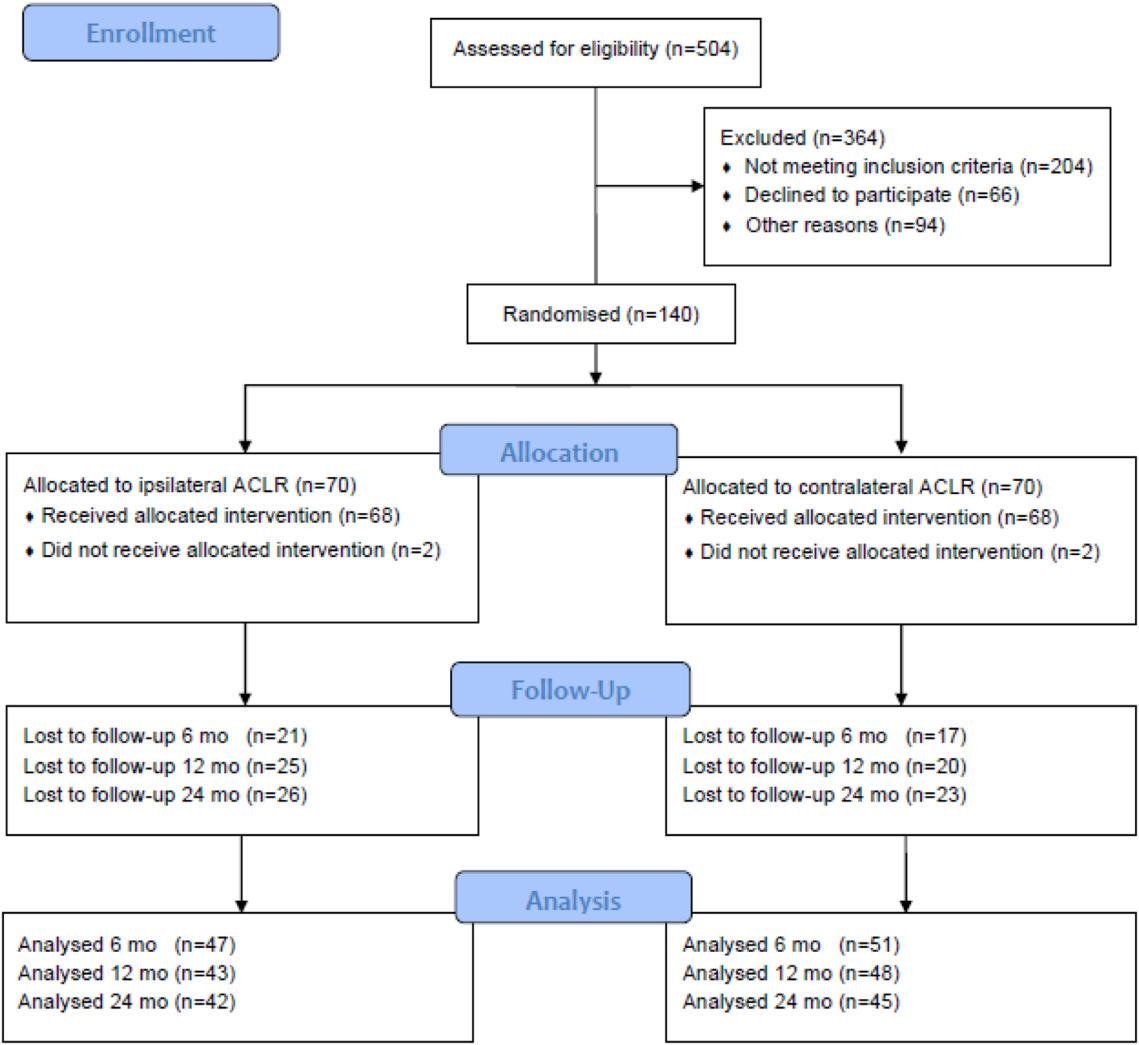
Enrollment and randomization of subjects

Between 2013 and 2017 an orthopedic research team assessed all patients presenting with an isolated ACL deficiency to the orthopedic outpatients clinic. Study eligibility was assessed according to inclusion and exclusion criteria, as listed in Table 1, with 140 of 504 patients deemed eligible, Fig. 1. All eligible patients received standardized information about the trial, orally and in writing, and informed consent was obtained from each patient prior to participation in the study. Randomization with the sealed envelope technique was performed by a research nurse.

**Table 1:** Inclusion and exclusion criteria

Inclusion criteria	Exclusion criteria
Unilateral ACL injury	Contralateral ACL injury
Age 18–50 years	PCL injury
	LCL injury
	MCL injury ≥ grade 2
	Multiligament injuries
	Significant hamstring injury

Patient demographics including age, gender, injured side, time from injury to surgery and concomitant injuries are presented in Table 2, with no significant differences between the groups.

**Table 2:** Descriptive study population

	Total (*n* = 137)	Ipsilateral ACLR, *n* = 68	Contralateral ACLR, *n* = 69	*P *value
Age at inclusion, mean ± SD	33.1 ± 9	33 ± 9	31.1 ± 9	n.s
Gender: female, *n* (%)	58 (42)	33 (48)	25(38)	n.s
BMI mean ± SD	25 ± 3	25 ± 4	25 ± 3	n.s
Type of activity when injured *n* (%)				n.s
Not specified	39 (28)	15 (22)	24(35)	
Soccer	32 (23)	15 (22)	17(25)	
Alpine ski/snowboard	18 (13)	11 (16)	7 (10)	
Indoor floorball	8 (6)	4 (6)	4 (6)	
MMA (mixed martial arts)	6 (4)	3 (4)	3 (4)	
Basketball	5 (4)	2 (3)	3 (4)	
Handball	3 (2)	1 (1)	2 (3)	
Badminton	3 (2)	2 (3)	1 (1)	
Work-related injury	3 (2)	2 (3)	1 (1)	
Am. Football	2 (1)	1 (1)	1 (1)	
Bandy	2 (1)	1 (1)	1 (1)	
Dance	2 (1)	1 (1)	1 (1)	
Gym	2 (1)	1 (1)	1 (1)	
Gymnastics	2 (1)	2 (3)	0	
Slipped on ice	2 (1)	2 (3)	0	
Horseback riding	2 (1)	0	2 (3)	
Beach volley ball	1 (1)	0	1 (1)	
Ice hockey	1 (1)	0	1 (1)	
Table tennis	1 (1)	0	1 (1)	
Trampoline	1 (1)	0	1 (1)	
Wakeboard	1 (1)	0	1 (1)	
Traffic accident	1 (1)	0	1 (1)	

Patient demographics at baseline for patients with an ACL tear are displayed as mean ± SD, number and percentage, respectively

Preoperative evaluation included measurements of instrumented laxity using a Rolimeter [6], thigh-circumference 10 cm proximal to the proximal pole of the patella, as well as a subjective and self-assessed Knee injury and osteoarthritis outcome score (KOOS) [19], IKDC [10], Lysholm score and Tegner activity level [28]. Tegner activity level prior to injury was also recorded. Follow-up examinations were performed at 6, 12, and 24 months postoperatively and included the same scores as preoperatively, as well as a functional strength test assessed with the single-leg hop.

Isokinetic peak torque strength at 60, 180 and 300°/s, and isometric torque strength at 60°, as well as total work in both extension and flexion was measured with Biodex^®^ [27] preoperatively and 6, 12 and 24 months postoperatively.

### Surgical technique

All ACLR were performed under general anesthesia by two experienced orthopedic surgeons, and apart from the harvesting site, the surgical procedure was identical for both groups.

After initial diagnostic arthroscopy, the tendons were harvested through a short, anteromedial oblique incision. If the single semitendinosus tendon was not sufficient in length and thickness, the gracilis tendon was harvested as well. The tendons were quadrupled over an adjustable loop Tightrope™ (Arthrex, Inc., Naples, FL, USA) and armed with nr.2 FibreWire™ (Arthrex, Inc., Naples, FL, USA). The femoral tunnel was drilled through an anteromedial portal after visualizing the anatomical insertion.

Tibial fixation was achieved by tibial TightRope ABS™ (Arthrex, Inc., Naples, FL, USA) suture to tension against the button for tibial cortical fixation.

### Post-operative management

The rehabilitation was standardized with full weight bearing allowed from day 1. Sports activities involving contact or pivoting moments were not permitted for 9 months post-operatively. Patients were permitted to choose rehabilitation center on their own, all familiar with the rehabilitation plan.

### Statistical analysis

Statistical analysis was performed with the IBM SPSS 25.0 software package for Macintosh. Nominal variables were tested by the χ^2^ test or the Fisher’s exact test. Ordinal variables and non-normally distributed interval and scale variables were evaluated by the Mann–Whitney *U* test, and the Student’s *t *test was used for normally distributed scale variables in independent groups. Longitudinal statistics were done with the paired-samples *t* test for normally distributed scale variables. Results were considered significant at *p* < 0.05.

A sample size calculation was performed using the primary endpoint isometric hamstring strength at 6 months. According to this calculation, if the mean difference is 10% or more and the common within-group standard deviation is 15, a sample size of 37 patients for the two groups will have a power of 80% to yield a statistically significant result with 5% risk of a type-one error.

## Results

### Demographics

Baseline demographics are presented in Table 3. The mean age of the study participants was 33.1 ± 9 years, with 58% male and 42% female patients. There were no significant differences in age, sex or additional injuries.

**Table 3:** Demographics

		Ipsilateral ACLR (*n* = 68)	Contralateral ACLR (*n* = 69)	*p *value
Time injury-recon	*d* ± SD	277 ± 277	179 ± 159	n.s
OP time	min ± SD	74 ± 15	83 ± 14	n.s
ST/G	*n* (%)	4 (6)	4 (6)	n.s
Additional injury	*n* (%)	41 (60)	41 (60)	n.s
Medial meniscus	*n* (%)	29 (42)	28 (40)	n.s
Lateral meniscus	*n* (%)	19 (28)	12 (17)	n.s
Sutures	*n* (%)	16 (24)	20 (29)	n.s
Suture medial	*n* (%)	11 (16)	12 (17)	
Suture lateral	*n* (%)	5 (7)	8 (12)	
Cartilage inj	*n* (%)	7 (10)	14 (20)	n.s

Reconstruction patient demographics at baseline for patients who underwent ACLR are displayed as mean ± SD, number and percentage, respectively

*ACL* anterior cruciate ligament

### Patient-related outcome

**Fig. 2:**
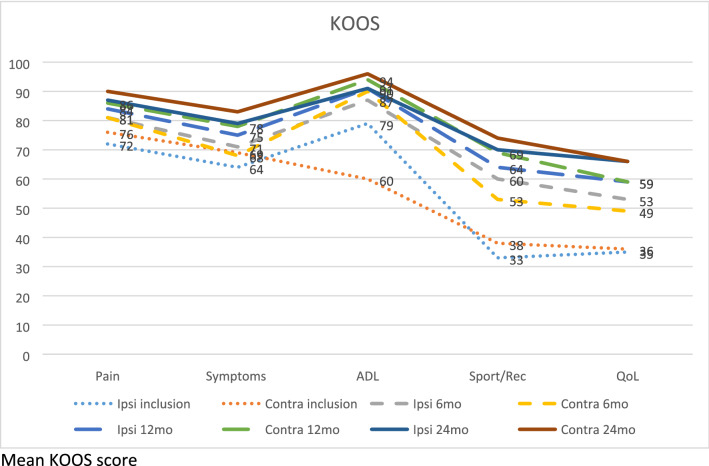
KOOS. mean KOOS score

As shown in Table 4 and Fig. 2, no difference in patient-related outcome scores were found. Lysholm [28], KOOS [19], IKDC [10], and Tegner [28] showed no statistically significant differences between the groups.

**Table 4:** Patient-reported outcomes

	Ipsilateral ACLR	Contralateral ACLR	*p*-value
Patient-reported outcomes at			
Lysholm mean (SD)^a^			
Inclusion (*n* = 64/65)	59 (16)	59 (14)	n.s
6 months (*n* = 61/62)	71 (15)	67 (15)	n.s
12 months (*n* = 53/56)	74 (16)	76 (14)	n.s
24 months (*n* = 53/64)	79 (20)	82 (15)	n.s
Tegner median (range)^b^			
Before injury	7 (2–10)	8 (4–10)	n.s
Inclusion	2 (0–7)	2 (0–9)	n.s
6 months	4 (0–10)	4 (0–8)	n.s
12 months	5 (0–10)	5 (0–9)	n.s
24 months	5 (0–10)	5 (2–10)	n.s
IKDC(SD)^c^			
Inclusion (*n* = 65/65)	51 (16)	52 (13)	n.s
6 months (*n* = 61/60)	60 (16)	57 (14)	n.s
12 months (*n* = 53/56)	70 (18)	74 (15)	n.s
24 months (*n* = 53/63)	72 (19)	75 (14)	n.s
FR *n* (%)^d^			
24 months	13 (19)	9 (13)	n.s
PASS KOOS *n *(%)^e^			
24 months	28 (52)	39 (61)	n.s
PASS IKDC *n* (%)^f^			
24 months	28 (52)	30 (48)	n.s
TF *n* (%)^g^			
24 months	8 (11)	8 (11)	n.s

*ACL *anterior cruciate ligament, *CL *uninjured contralateral limb

^a^Score range from 0 to 100, with higher scores indicating better results

^b^Assesses activity level with specific emphasis on knee; scores range from 1 (least strenuous activity) to 10 (high knee demanding activity on professional sports level)

^c^Score range from 0 to 100, with higher scores indicating better results

^d^Defined as knee osteoarthritis outcome score (KOOS) above: 90 for pain, 84 for symptoms, 91 for ADL, 80 for Sport/Rec and 81 for quality of life (QoL)

^e^defined as Knee Osteoarthritis Outcome Score (KOOS) above 88.9 for Pain, 57.1 for Symptoms, 100.0 for ADL, 75.0 for the Sport/Rec, and 62.5 for QoL

^f^Defined as IKDC score > 75.9

^g^Defined as KOOS, QoL < 44

### Functional recovery (FR), Patient acceptable symptom state (PASS) and treatment failure (TF)

FR is defined as a Knee Osteoarthritis Outcome Score (KOOS) above: 90 for Pain, 84 for Symptoms, 91 ADL, 80 for Sport/Rec and 81 for quality of life (QoL), while the PASS thresholds for IKDC score is above 75.9 and for KOOS they are 88.9 for Pain, 57.1 for Symptoms, 100.0 for ADL, 75.0 for the Sport/Rec, and 62.5 for QoL [7, 18]. TF is defined as a KOOS, QoL < 44 [7]. No significant differences between the groups were found Table 4.

### Objective measures

ROM as well as manual laxity measurements did not demonstrate any significant differences between the IL and CL surgery patients, Table 5.

**Table 5:** Objective measures

	Ipsilateral ACLR	Contralateral ACLR	*p *value
Instrumented knee laxity			
Rolimeter mean mm (SD)			
6 months (*n* = 42/47)	1.3 (1.3)	1.3 (1.2)	n.s
12 months (*n* = 39/41)	1.4 (1.5)	2.0 (3.1)	n.s
24 months (*n* = 45/47)	1.7 (1.8)	1.5 (1.5)	n.s
Range of motion			
Ext. def > 5° compared to CL *n* (%)			
6 weeks (*n* = 70/65)	10 (14)	5 (8)	n.s
6 months (*n* = 46/48)	4 (8)	7 (14)	n.s
12 months (*n* = 42/51)	3 (7)	7 (13)	n.s
24 months (*n* = 48/49)	4 (8)	4 (8)	n.s
No (%) normal Pivot Shift test^a^			
24 months (*n* = 48/50)	43 (90)	48 (96)	n.s
No (%) normal Lachmann test^b^			
24 months (*n* = 48/50)	48 (100)	49 (98)	n.s
Functional strength			
Thigh deficit circ. 10 cm above patella diff in cm ref CL			
6 months (*n* = 51/53)	1 (1)	1 (1)	n.s
12 months (*n* = 46/51)	1 (1)	0 (1)	0.04
24 months (*n* = 48/50)	0 (1)	0 (1)	n.s
One leg hop *n*(%)^c^			
6 months (*n* = 40/44)			
> 90	16 (40)	19 (43)	n.s
76–89	14 (35)	12 (27)	
50–75	6 (15)	9 (21)	
< 50	4 (10)	4 (9)	
12 months (*n* = 34/47)			
> 90	19 (56)	29 (62)	n.s
76–89	6 (18)	14 (30)	
50–75	5 (15)	3 (6)	
< 50	4 (12)	1 (2)	
24 months (*n* = 44/46)			
> 90	27 (61)	31 (67)	n.s
76–89	14 (32)	9 (20)	
50–75	3 (7)	2 (4)	
< 50	0	4 (9)	

*ACLR* anterior cruciate ligament reconstruction, *CL *contralateral limb

^a^Assesses rotational stability of knee at rest result range from 0 (normal stability) to 3 (severely increased instability)

^b^Assesses rotational stability of knee at rest result range from 0 (normal stability) to 3 (severely increased instability)

^c^Result indicates if the patient is ready to return to play, to pass, the involved leg must measure at least 90% of the distance compared to the uninvolved leg

Similar results were found in the groups regarding muscle circumference and functional strength measured with the one-leg hop test.

### Functional strength

The CL group demonstrated significantly stronger isometric extension muscle strength at both 6 and 12 months, but not at 24 months.

Regarding isokinetic flexion muscle strength and total work in flexion, the IL group were significantly weaker in all velocities during the trial period.

**Fig. 3:**
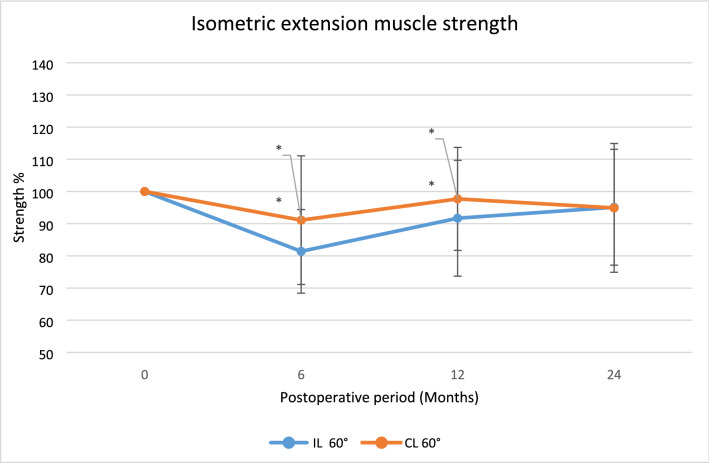
The average isometric extension muscle strength after surgery displayed as mean percentage with reference contralateral leg set at 100,*p < 0.05

**Fig. 4:**
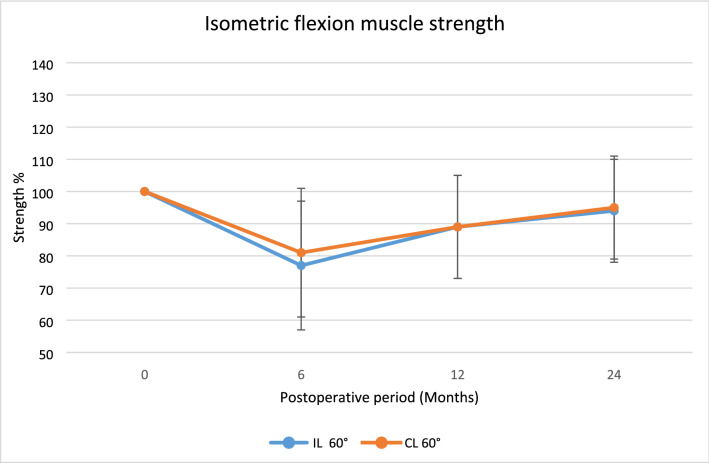
The average isometric lexion muscle strength after surgery displayed as mean percentage with reference contralateral leg set at 100, *p < 0.05

**Fig. 5:**
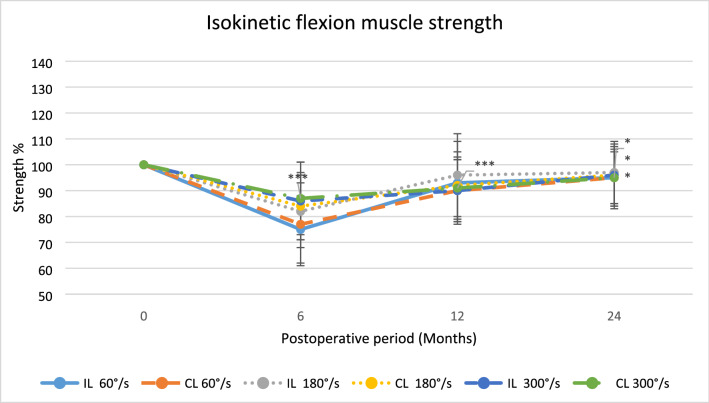
The average isokinetic flexion muscle strength after surgery displayed as mean percentage with reference contralateral leg set at 100,*p < 0.05

**Fig. 6:**
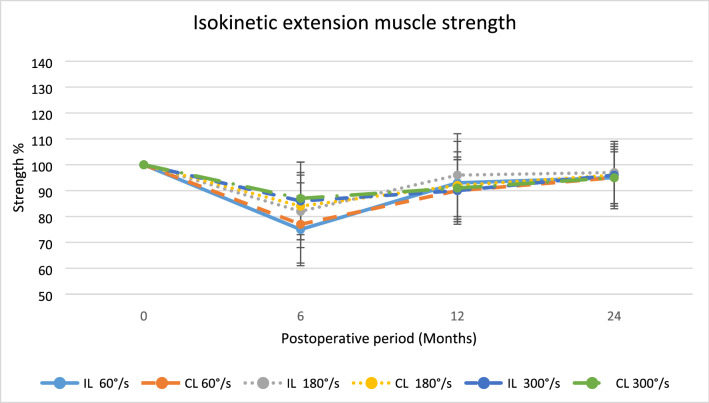
The average isokinetic ext. muscle strength after surgery displayed as mean percentage with reference contralateral leg set at 100, *p < 0.05

**Fig. 7:**
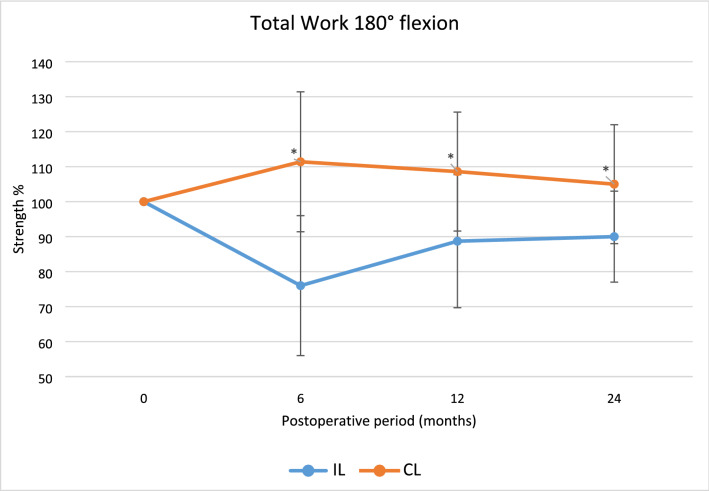
The average total work at 180°/s after surgery displayed as mean percentage with reference contralateral leg set at 100, *p < 0.05

There were no statistically significant differences in any other strength assessments, although higher values were found for the CL group, Figs. 3, 4, 5, 6, 7.

### Additional surgery

Additional surgery was required in nine cases (13%) in the IL group and 15 (21%) in the CL group (n.s.), Table 6. Two patients in each group sustained a graft rupture during the study period and both reported a significant new trauma. One patient in the CL group suffered a contralateral ACL rupture.

**Table 6:** Additional surgery

	Ipsilateral ACLR (*n* = 68)	Contralateral ACLR (*n* = 68)	*p *value
Additional surgery within 24 months, *n* patients (%)	9 (13)	15 (21)	n.s
Reason for reoperation *n* (%)			
Cyclops lesion	4 (6)	8 (11)	
Graft rupture	2 (3)	2 (3)	
Endobutton removal	0	1 (1)	
Contralateral ACLR	0	1 (1)	
Meniscal lesion	1 (1)	3 (4)	
Infection	2 (3)	0	

## Discussion

The most important finding of the present study was that the use of a contralateral ST graft facilitates earlier isokinetic and isometric strength recovery after ACLR.

The results support the hypothesis that the use of a contralateral ST graft in ACLR, in comparison to an ipsilateral ST graft, can improve muscle strength in knee flexion and facilitate early symmetrical strength between limbs. A further finding was that both groups demonstrated improvements in all self-reported and objective assessments up to one-year post surgery. These improvements plateaued from 12 to 24 months, with no significant differences observed between the groups.

This study found a significant deficit in both isometric and isokinetic flexion strength in the limb where the graft was harvested. This reflects the findings of other studies [5, 14, 15, 31] which have shown continued strength deficits more than 2 years post-surgery. In contrast, previous studies by Yasuda et al. and McRae et al. [16, 33] did not find any long-term differences between limbs.

In this study, symmetrical leg strength was achieved as early as 6 months postoperatively in the CL group, however, in the IL group isokinetic flexion strength remained asymmetrical for the duration of the trial.

There was no difference between the groups with respect to IKDC, Lysholm, KOOS or knee laxity. This is in line with earlier studies by Yasuda and McRae [16, 33]. Tegner level did not return to preinjury level. It is unclear if this was due to knee function or other factors. A contributing factor may have been the mean age of study participants, 33, an age where family and career commitments may be demanding, potentially resulting in lower activity levels. A further consideration is that although this study was initially designed to only include highly active patients, those with lower demands as well as non-active patients and patients with an unstable knee were also included. This may have contributed to the continued muscle weakness recorded at 24 months follow-up. In a meta-analysis Ardern et al. [4] reported that two-thirds of patients manage to return to their previous activity level and 82% returned to some type of sport participation. Von Essen et al. [29] reported from the same institute a return to pre-injury at 86%, however, the participants in that study were highly active athletes with a strong desire to return to sport. This study also shows the difference between FR and PASS and why these values are different and not interchangeable. While FR is equal to a return to an almost pre-injury KOOS level, PASS is a measure of what the patient finds in an acceptable state. FR is only half of what Barenius et al. [7] found, while PASS values are in line with other studies [8, 11]. TF is also only a third of what Barenius et al. found, and in line with Ingelsrud et al. although their cut off was set lower [7, 11]. These patients might not be struggling to get back to high-level sports, instead they are happy with a functional knee that does not give away.

A theoretical advantage of CL graft harvest is that the ST tendon’s contribution to dynamic stabilization is not compromised, hence normal knee biomechanics are better maintained. However, if the contralateral healthy knee develops impaired knee kinematics as a result of graft harvesting, this could also be a potential disadvantage which could increase the risk of injury. Previous studies have found the risk of a contralateral ACL rupture is approximately 3% [3, 13]. Andernord et al. [3] found in the Swedish national anterior cruciate ligament register that females undergoing ACLR have a 3 times higher risk of ACL rupture on the contralateral side following CL harvest. According to these figures the number of CL ruptures in this study should have been 1.8 (3%) for the CL group and for the 23 females in CL group the expected numbers should be 1.9(9%), however, only one was observed. Although not adequately powered to address this issue conclusively, an increased rate of CL graft ruptures was not apparent in this study, hence CL graft harvest does not appear to increase the risk of reinjuries or ruptures of the contralateral ACL. A longer follow-up period would allow us to better evaluate this risk.

The major strength of this study is the prospective, randomized design with two experienced orthopedic surgeons performing the ACLR with the same standardized technique. The groups were comparable in terms of age, gender and additional injuries and both subjective and objective measurements were made.

There are, however, limitations for this study. Firstly, rehabilitation was not made at the same center and there was no measurement of compliance to the standardized rehabilitation protocol that was provided.

Another weakness of this study was a loss to follow-up. It was anticipated some loss to follow up and therefore enrolled 140 patients instead of the stipulated 94, which would have been sufficient with a 20% loss to follow up, however, this figure was exceeded with regard to some evaluations. The smaller sample size may have reduced the power of the study, however, an adequate number of patients were nonetheless analyzed according to the pre-study power analysis.

## Conclusion

This study demonstrates that utilizing a ST graft harvested from the uninjured limb for ACLR facilitates early isokinetic and isometric strength recovery, with no significant adverse outcomes demonstrated in other measurements and therefore be performed to reduce the risk of long-term strength deficits in the injured leg.
